# Gut microbial biomarkers for the treatment response in first-episode, drug-naïve schizophrenia: a 24-week follow-up study

**DOI:** 10.1038/s41398-021-01531-3

**Published:** 2021-08-10

**Authors:** Xiuxia Yuan, Yunpeng Wang, Xue Li, Jiajun Jiang, Yulin Kang, Lijuan Pang, Peifen Zhang, Ang Li, Luxian Lv, Ole A. Andreassen, Xiaoduo Fan, Shaohua Hu, Xueqin Song

**Affiliations:** 1 Department of Psychiatry, The First Affiliated Hospital/Zhengzhou University, Zhengzhou, China; 2 Biological Psychiatry International Joint Laboratory of Henan/Zhengzhou University, Zhengzhou, China; 3 Henan Psychiatric Transformation Research Key Laboratory/Zhengzhou University, Zhengzhou, China; 4 Centre for Lifespan Changes in Brain and Cognition (LCBC), Department of Psychology, University of Oslo, Forskningsveien 3A, 0373 Oslo, Norway; 5 Department of Psychiatry, The First Affiliated Hospital, Zhejiang University School of Medicine, Hangzhou, China; 6 Chinese Research Academy of Environmental Sciences, Institute of Environmental Information, Beijing, China; 7 Henan Gene Hospital, The First Affiliated Hospital of Zhengzhou University, Zhengzhou, China; 8 Henan Province Mental Hospital, The Second Affiliated Hospital/ Xinxiang Medical University, Xinxiang, China; 9 Norwegian Centre for Mental Disorders Research (NORMENT), Institute of Clinical Medicine, University of Oslo, and Oslo University Hospital, Kirkeveien 166, 0450 Oslo, Norway; 10 Psychotic Disorders Program, UMass Memorial Medical Center/University of Massachusetts Medical School, Worcester, MA USA

**Keywords:** Schizophrenia, Human behaviour

## Abstract

Preclinical studies have shown that the gut microbiota can play a role in schizophrenia (SCH) pathogenesis via the gut-brain axis. However, its role in the antipsychotic treatment response is unclear. Here, we present a 24-week follow-up study to identify gut microbial biomarkers for SCH diagnosis and treatment response, using a sample of 107 first-episode, drug-naïve SCH patients, and 107 healthy controls (HCs). We collected biological samples at baseline (all participants) and follow-up time points after risperidone treatment (SCH patients). Treatment response was assessed using the Positive and Negative Symptoms Scale total (PANSS-T) score. False discovery rate was used to correct for multiple testing. We found that SCH patients showed lower α-diversity (the Shannon and Simpson’s indices) compared to HCs at baseline (*p* = 1.21 × 10^−9^, 1.23 × 10^−8^, respectively). We also found a significant difference in β-diversity between SCH patients and HCs (*p* = 0.001). At baseline, using microbes that showed different abundance between patients and controls as predictors, a prediction model can distinguish patients from HCs with an area under the curve (AUC) of 0.867. In SCH patients, after 24 weeks of risperidone treatment, we observed an increase of α-diversity toward the basal level of HCs. At the genus level, we observed decreased abundance of *Lachnoclostridium* (*p* = 0.019) and increased abundance *Romboutsia* (*p* = 0.067). Moreover, the treatment response in SCH patients was significantly associated with the basal levels of *Lachnoclostridium* and *Romboutsia* (*p* = 0.005 and 0.006, respectively). Our results suggest that SCH patients may present characteristic microbiota, and certain microbiota biomarkers may predict treatment response in this patient population.

## Introduction

Schizophrenia (SCH) is a chronic and severe mental disorder, characterized by impaired cognitive functions and social disability [1]. Despite that SCH incurs a heavy burden on family and society, the etiology of the disease is largely unknown [2, 3]. Preclinical research has suggested that the dysbiosis of gut microbiota could perturb several pathophysiological pathways thus contributing to the risk of SCH, e.g., disruption of glutamatergic neurotransmission and the glutamate-glutamine-GABA (γ-aminobutyric acid) cycle [4, 5], and dysregulation of tryptophan’s metabolites and neuroimmune activation [6–8]. It was also suggested that the immune and inflammatory mechanisms may be the link between the gut microbiota and the risk of SCH [9]. The SCH-enriched bacteria recipient mice showed deficits in social behaviors and changes in the expression of immune/inflammation-related genes in the intestine, suggesting that dysfunction of the gut microbiota may affect the functioning of inflammation pathways and schizophrenia-like behavior in the animals [10]. However, the clinical implications of these preclinical findings are still unclear.

The associations between the gut microbial compositions and the risk of SCH have also been reported in several psychiatric patient studies [5, 9–13]. The alteration of phylum especially *Firmicutes* was reported to be associated with psychiatric disorders, e.g., schizophrenia [11, 12, 14], depression [15, 16], and autism [17], although specific findings vary across different studies. Oropharyngeal microbiome analysis has shown that SCH patients had a higher abundance of *Firmicutes* but the lower abundance of *Bacteroidetes* and *Actinobacteria* compared to healthy controls (HCs) [11]; intestinal microbiome analysis has found a higher abundance of phylum *Proteobacteria* [12] and *Actinobacteria* [9, 14] but the lower abundance of *Firmicutes* [12, 14] in SCH patients compared to HCs. Genus analysis has shown that some genera from *Firmicutes* showed lower levels in SCH patients than in HCs, such as *Blautia*, *Coprococcus*, *Roseburia*, *Blautia*, *Streptococcus*, *Enterococcus* [12, 13]; other genera from *Firmicutes*, including *Megasphaera*, *Lactobacillus*, and *Clostridium*, were enriched in SCH [12, 13]. In addition, several genera from the phyla *Proteobacteria* [12] (e.g., *Succinivibrio*, and *Klebsiella*) and *Actinobacteria* [9, 12, 18, 19] (e.g., *Collinsella*, *Actinomyces*, and *Eggerthella*) showed higher levels in SCH patients. Albeit inconsistent associations for specific microbial taxa, these studies indicated that patients with SCH displayed a dysbiosis of gut microbiota. Moreover, microbial biomarkers belonging to phylum *Firmicutes*, such as genera *Lactobacillus*, family *Lachnospiraceae*, *Ruminococcaceae*, *Veillonellaceae*, and *Streptococcaceae* were reported to be significantly correlated with symptom severity [5, 13, 20]. However, the role of gut microbiota in treatment response for SCH remains unclear.

Here, we present a 24-week study, including 107 first-episode, drug-naïve SCH patients, and 107 matched HCs. SCH patients were treated with risperidone monotherapy. Our primary goal was to identify the gut microbial biomarkers associated with the risperidone treatment response. We also would like to characterize microbiota in SCH patients. Inflammatory markers, hypersensitive C-reactive protein (hs-CRP), and homocysteine (HCY) (HCY-induced oxidative stress and inflammation via Nox4/NF-κB pathway) [21] have been reported to be associated with SCH [22, 23], we additionally explored the relationship of gut microbiota, inflammatory markers, and treatment response.

## Methods

This study was approved by the Human Ethics Committee of the First Affiliated Hospital of the Zhengzhou University, China (Approval No. 2016-LW-17). All enrolled first-episode, drug-naïve SCH patients have been carefully assessed by a psychiatrist between October 2017 and 2019. The following inclusion criteria were used: (1) diagnosis of SCH based on the Diagnostic and Statistical Manual of Mental Disorders fourth version (DSM-IV) criteria, confirmed by the Structured Clinical Interview for DSM-IV (SCID) [24]; (2) medication free; (3) a Positive and Negative Syndrome Scale (PANSS) total score >60. Patients with the following conditions were excluded: (1) diagnosis of autoimmune diseases, heart diseases, hepatobiliary and gastrointestinal diseases, blood diseases, diabetes (type I and type II), neurological diseases, mental retardation, or other psychiatric diseases; (2) being pregnant or lactating; (3) treated with any antibiotics or anti-inflammatory agents in the previous month; (4) being obese (body mass index, BMI > 28 kg/m^2^) [25]. HCs were recruited from local communities through an online advertisement. HCs were matched to SCH patients by age, gender, education, smoking habits, and BMI. The same exclusion criteria applied to SCH patients were applied to HCs. In total, 107 patients and 107 HCs at baseline were included (Supplementary Figure 1). All subjects provided signed informed consent.

### Assessments

After admission to the hospital, the clinical assessment was performed, and biological samples were collected before risperidone treatment. All subjects completed a comprehensive clinical interview, including the SCID, and provided information on age, gender, education, smoking, disease duration, and medication history. Educations were calculated based on schooling years (including expected years of education, i.e., from the year of enrollment to the year of expected completion of education). Disease duration (days) was calculated as the time from the first appearance of the psychiatric symptoms to the first hospitalization. Before (week 0; S0), during (week 6 and 12; S1 and S2), and after (week 24; S3) treatment, study participants were assessed with PANSS, and side effects and somatic health screening. For SCH patients, the PANSS was performed at baseline, week 6, week 12, and week 24. The PANSS total scores (PANSS-T) include positive symptoms scores (PANSS-P), negative symptoms scores (PANSS-N), and general psychopathology symptoms scores (PANSS-G). PANSS factor scores for negative symptoms (PANSS-FSNS) and positive symptoms (PANSS-FSPS) were calculated as previously described [26]. The treatment response was defined as the mean change of PANSS-T scores from baseline to week 24.

### Antipsychotic treatment

Weighing the efficacy and side effects of antipsychotics [27] and clinical experience, risperidone (dopamine D_2_ receptor and 5-HT_2_ receptor antagonism) was used for pharmacological treatment. The dosage of risperidone was gradually titrated from 1 mg/day to 4–6 mg/day as clinically indicated. Clonazepam was used in three patients who had sleep problems. Eight patients had an extracorporeal vertebral response after risperidone was titrated to 6 mg/d, and benzyl was used to improve the conditions. For various reasons, not all patients completed the full study. In the 6th-week, five patients did not provide stool samples; five patients did not come to our hospital due to unknown reasons, and one dropped due to hospitalization for poor treatment response. For the assessment at the 12th week, 10 patients did not come to our hospital due to unknown reasons, and 12 patients did not provide stool samples. In the 24th week, four patients did not come to our hospital, and four patients stopped medicine due to economic reasons. In total, 60 SCH patients completed the 24-week study (Supplementary Figure 1).

### Biological samples collection

Fresh fecal samples were collected at baseline, week 6, week 12, week 24, and stored at a −80 °C refrigerator immediately for the gut microbiota assay. The blood samples were collected at the same time for measuring inflammatory parameters, including hs-CRP and HCY. Serum levels of HCY were detected using an immune turbidimetric test (Roche Cobas c501, Switzerland). Serum levels of hs-CRP were measured by a chemical colorimetry assay and a particle-enhanced immunoturbidimetric assay respectively using a Roche automatic biochemical analyzer (Roche Diagnostics, C8000, Germany). All assays were performed according to the manufacturer’s instructions. The gut microbiota and inflammatory biomarkers were measured with the subject’s condition (patients vs. controls) blinded.

### DNA extraction

DNA was extracted from 0.2 g of the fecal sample using the Cetyltrimethylammonium Ammonium Bromide CTAB/SDS method [28]. The purity and concentration of DNA were monitored using gel electrophoresis on 1% agarose gels. An amount of 150 μl of the DNA sample was obtained in the centrifuge tube and was diluted with sterile water to 1 ng/μl.

### Amplicon generation

The 16 S rRNA gene of distinct regions (V3-V4) was amplified using a specific primer with barcodes. The individually barcoded 341 F forward primers and the 806 R reverse primers were CCTAYGGGRBGCASCAG and GGACTACNNGGGTATCTAAT, respectively. All PCRs were performed as 30 μL reactions with 15 μL of Phusion® High-Fidelity PCR Master Mix (New England Biolabs), and 0.2 μM of forward and reverse primers and 10 ng of template DNA. The reaction cycles consisted of 98 °C pre-denaturation for 1 min, followed by 30 cycles of 98 °C denaturation for 10 s, annealing at 50 °C for 30 s, elongation at 72 °C for 30 s, and 72 °C elongation for 5 min.

### PCR products purification

After electrophoresis on 2% agarose gel, PCR products were purified using GeneJETTM Gel Extraction Kit (Thermo Scientific) and quantified according to the manufacturer’s protocol and multiplexed at equal concentrations.

### Library preparation and sequencing

The resultant library was sequenced on the Ion S5 TM XL platform and 400 bp/600 bp single-end reads were generated. Raw DNA sequences were quality controlled (QC) using Cutadapt (V1.9.1, http://cutadapt.readthedocs.io/en/stable/) [29]. Chimera sequences [30] were removed by comparing DNA sequences with the Silva reference database [31] using the UCHIME algorithm [32].

### Bioinformatic analysis

Sequence analyses were performed using the Uparse software (V7.0.1001) [33]. A widely employed similarity threshold of 97% was used to assign quality-controlled sequences to operational taxonomic units (OTUs). The taxonomy of microbes was determined using the Mothur algorithm (https://www.mothur.org) based on the Silva Database (https://www.arb-silva.de/) [31].

α-diversity was estimated using the Shannon index, Simpson index, Polygenetic Diversity Index (PD whole tree), Observed Species index, Chao index, and Abundance Coverage-based Estimator (ACE index) using QIIME (V1.7.0). β-diversity was computed based on the Bray Curtis Dissimilarity, Binary Jaccard Dissimilarity, Weighted and Unweighted UniFrac Distance. the former two calculation does not consider phylogenetic relatedness of taxa but the latter two calculation does. The difference in β-diversity between SCH patients and HCs was examined using permutational multivariate analysis of variance (PERMANOVA). Principal Coordinate Analysis (PCoA) was performed to obtain principal coordinates. PERMANOVA was conducted using the vegan package of the R software (V3.6.2). PCoA results were displayed using the WGCNA package, the stat packages, and the ggplot2 package of the R software (V3.6.2).

Microbial Dysbiosis Index (MD index) was determined using log (A/B), where A is the total abundance of genera that was higher in SCH patients than in HCs; B is the total abundance of genera that was lower in SCH patients than in HCs [34].

### Statistical analyses

For continuous variables, within each group, a normality test was performed using the Shapiro–Wilk test, the homogeneity of variance was performed using Levene’s test. For categorical variables, the Chi-squared test was used to check for independence among variables. In all statistical analyses, age, gender, education, disease duration, smoking status, and BMI were used as covariates. False discovery rate (FDR) [35] was used to correct multiple testing in the present study.

### Differences in microbial biomarkers between SCH patients and HCs at baseline

The Wilcoxon test was used to identify differential abundant bacteria between patients and HCs. Microbial biomarkers that have a mean abundance ≥0.001 and ≥60% of samples with the abundance of microbial biomarkers ≥0.001, were analyzed in the present study.

The Random Forest methods [36] (randomForest package [37] from R V3.6.2) were used to construct prediction models for SCH using differential abundant microbial biomarkers between patients and HCs. A cross-validation procedure was employed to evaluate the generalization performance of the resultant prediction model. Two-thirds of the samples were randomly selected as training set to build the prediction models; the remaining 1/3 samples were used as the verification set. This procedure was repeated 1000 times, and the average values for the performance metrics were used as indicators for the predictive performance of the model. Gini importance (or mean decrease impurity) is computed from the Random Forest method to describe which features are relevant. The receiver operating characteristic (ROC) curve function from the randomForest package was used to compute the area under the curve (AUC).

### Changes in gut microbiota and clinical symptoms after risperidone treatments in SCH patients

The linear discriminant analysis (LDA) effect-size (LEfSe) method was used to perform metagenomics analysis [38, 39]. LDA was used to reduce the dimensionality of the data and evaluate the influence of the statistically different microbes (LDA score). The default parameters of the LEfSe package were used, alpha = 0.05 and effect-size threshold = 3.0. The paired Wilcoxon test was used to test the changes in the differential abundant bacteria, which were identified at baseline in SCH patients, at different time points after risperidone treatment. A similar procedure was applied for the PANSS scores and inflammatory biomarker hs-CRP and HCY measured in this study.

### Correlations between microbial biomarkers and the PANSS total scores

The partial correlations between the severity of symptoms and microbial biomarkers that were different between SCH and HCs at baseline and/or significantly changed after risperidone treatment in patients were also analyzed after controlling for various covariates.

### Relationship between the changes in the PANSS total scores and the changes in microbial biomarkers

The relationship between the changes of the PANSS total scores and the changes in microbial biomarkers that were different between SCH and HCs at baseline and/or significantly changed after risperidone treatment were examined using stepwise linear regression models using the *F* test with parameters, *p* value threshold in = 0.05 and threshold out = 0.10. The changes in the PANSS total scores were set as dependent variables, the changes in microbial biomarkers and inflammatory biomarkers (hs-CRP and HCY), and various covariates were set as independent variables.

### Baseline microbial biomarker levels and treatment response in SCH patient

The relationship between the treatment response and the gut microbial biomarkers that were different between SCH and HCs at baseline and/or significantly changed at different time points in patients were explored using random-intercept linear mixed-effect models. The changes of the PANSS total scores at each time point were set as dependent variables and the time point, baseline microbial biomarker levels, and various covariates were set as fixed-effect predictors. The participant identifiers were used as a random variable in the models. The lme4 [40] and the lmeTest packages from R (V3.6.2) were used for these analyses. For mixed-effect models, the approximated *p* values <0.05 were considered statistically significant.

## Results

### Clinical characteristics and evaluation

There were no significant differences in age, gender, education, smoking, and BMI between the SCH patients and HCs (all *p* > 0.05) (Table 1**)**. We observed higher levels of hs-CRP and HCY in the SCH group than in HCs at baseline (*p* = 0.035, 1.49 × 10^−6^, respectively) (Table 1).

**Table 1:** Demographic characteristics and statistical analysis.

	SCH patients (*N* = 107)	Healthy controls (*N* = 107)	*Z*, *t*, or *X*^2^ value	*p* value
	Median (IOR)	Median (IOR)		
Age (years)	19 (19–25)	23.00 (22.00–25.00)	1.642	0.103
Education years	12 (11–13)	12 (12–12)	−0.654	0.513
Body weight (kg)	58.6 (51–65)	57.15 (52.3–67.5)	−0.579	0.563
BMI (kg/m^2^)	20.71 (18.91–22.41)	21.17 (19.41–23.16)	−1.373	0.170
hs-CRP (mg/L)	0.28 (0.15–0.62)	0.20 (0.13–0.40)	−1.995	0.035
HCY (mmol/L)	14.81 (12.10–19.50)	10.45 (8.36–17.86)	−3.882	1.49 × 10^−6^
PANSS-FSPS	17.00 (13.75–19.00)			
PANSS-FSNS	19.00 (14.75–23.00)			
PANSS-G	39.00 (35.00–47.00)			
PANSS-T	79.50 (72.00–89.25)			
Disease duration	180 (30–365)			
Gender				
Male	51 (48)	37 (35)	3.783	0.071
Female	56 (52)	70 (65)		
Smoking				
Yes	5 (5%)	6 (6%)	0.096	0.500
No	102 (95%)	101 (94%)		

Note: *Hs-CRP* high-sensitivity C-reactive protein, *HCY* homocysteine, *PANSS-FSPS* PANSS factor score for positive symptoms, *PANSS-FSNS* PANSS factor score for negative symptoms, *PANSS* The Positive and Negative Syndrome Scale, including positive, negative, and general psychopathology symptoms represented by PANSS-P, PANSS-N, and PANSS-G, respectively, PANSS-T=PANSS-P+PANSS-N+PANSS-G, *SCH* schizophrenia.

**Fig. 1:**
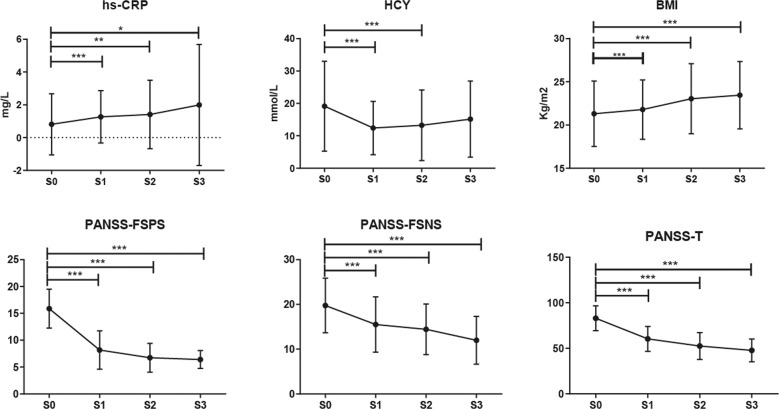
The dynamic changes of inflammatory biomarkers and clinical improvements. The dynamic changes of BMI, inflammatory biomarkers hs-CRP and HCY in SCH patients. PANSS scores show a significant decrease after risperidone treatment. *p < 0.05, **p < 0.01, ***p < 0. 001. The means and standard deviation are indicated in the bar chart. FDR were used to correct the multiple testing.

After 24 weeks of risperidone treatment, serum HCY levels decreased and hs-CRP and BMI increased significantly (all *p* < 0.05, Fig. 1). In addition, clinical improvement was observed as reflected by the reduction in the PANSS scores (all *p* < 0.05, Fig. 1).

After 24 weeks of treatment, the baseline levels of hs-CRP were positively correlated with the changes of the PANSS-T scores (*p* = 0.015). The baseline levels of HCY were also positively correlated with the changes of the PANSS-FSPS (*p* = 0.012).

### Microbial biomarker annotation

In total, 472 samples from all recruited participants were sequenced using IonS5^TM^XL sequencer platform. From 472 samples, 76,747 high-quality reads were obtained. These reads were clustered into 8450 qualified OTUs at 97% sequence similarity and 8433 (99.80%) of them could be annotated to the database. 95.17, 84.53, and 61.51% of all reads were annotated to phylum, family, and genus. In order to ensure the accuracy and reliability of sequencing data, 28 duplicated samples were randomly selected and sequenced. Paired sample *T*-test showed no significant differences in the diversity and composition of intestinal microorganisms between the two groups of samples (Supplementary Table 1). Finally, 444 samples were further analyzed.

A Venn diagram showed that 2223 of 5416 OTUs were detected in the two groups, while 2270 and 923 OTUs were unique to patients with SCH and HCs, respectively (Supplementary Figure 2A). Most rarefaction curves tended to approach the saturation plateau, suggesting that the sequencing depth was enough to cover the whole bacterial diversity (Supplementary Figure 2B). The box plot tended to approach the saturation plateau, indicating that the sample capacity was sufficient for data analysis (Supplementary Figure 2C). The predominant phylum and genus bacteria in SCH and HCs group were shown in Supplementary Figure 2D, E.

### Change in microbial α-diversity in SCH patients

**Fig. 2:**
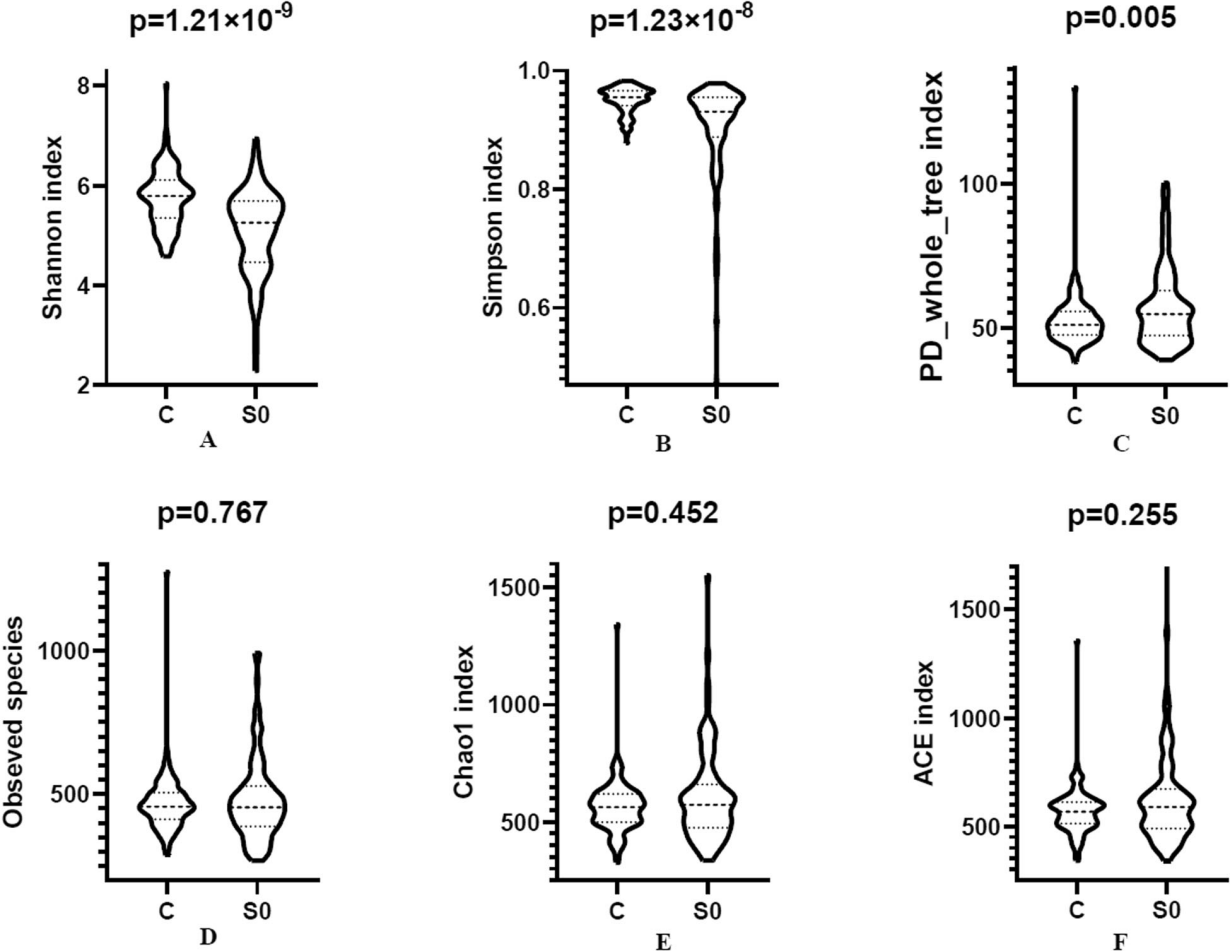
The alteration of α diversity in SCH patients. S0: schizophrenia, C: healthy controls. FDR were used to correct the multiple testing.

We analyzed six α-diversity indices of SCH patients and HCs based on the OTU relative table. We analyzed Shannon index and Simpson index, which reflect richness and evenness. We found significant lover levels of Shannon and Simpson’s indices in SCH patients than in HCs (*p* = 1.21 × 10^−9^, 1.23 × 10^−8^, respectively) (Fig. 2A, B). PD whole tree index incorporates phylogenetic differences, and the phylogenetic diversity reflects the phylogenetic distance of each species in a community. The more complex the species’ kinship within the community is, the farther the evolutionary distance and the higher levels of phylogenetic diversity are. When using phylogenetic diversity, we observed a significant increase in this measure in the SCH patients than in HCs (Fig. 2C). Both observed species index and Chao1 index reflect species richness; the Chao1 index can reflect the existence of low abundance species in the community. Abundance Coverage-based Estimator (ACE) reflects the total number of species, considering both species abundance and the probability of species in the sample. In these measures, there were no significant differences between SCH patients and HCs (Fig. 2D–F). Our result suggests that the internal similarity of the bacterial community in SCH patients is higher than in HCs.

### Change in microbial β-diversity in SCH patients

We then analyzed the β-diversity to evaluate whether the whole microbiome community was different between SCH patients and HCs. We found a significant difference in bacterial community composition between SCH patients and HCs based on Bray-Curtis and Binary Jaccard dissimilarity calculations, which do not take phylogenetic relatedness of taxa into consideration (Supplementary Figure 3A, B). Similarly, there was a significant difference in the composition of bacterial gut microbiota based on weighted and unweighted UniFrac distances, which take phylogenetic relatedness into account (Supplementary Figure 3C, D). The PERMANOVA based on those four β-diversity dissimilarity metrics shows that the between-group difference was significantly different than the within-group difference, in either SCH patients or HCs (all *R*^2^ > 0, *p* < 0.05) (Supplementary Table 2); accordingly, Principal Coordinate Analysis (PCoA) based on the four metrics showed a significant separation of the two groups (Supplementary Figure 4A–D). These results indicating a significant difference in bacterial community composition between SCH patients and HCs.

### Dysbiosis of gut microbiota in SCH patients

**Fig. 3:**
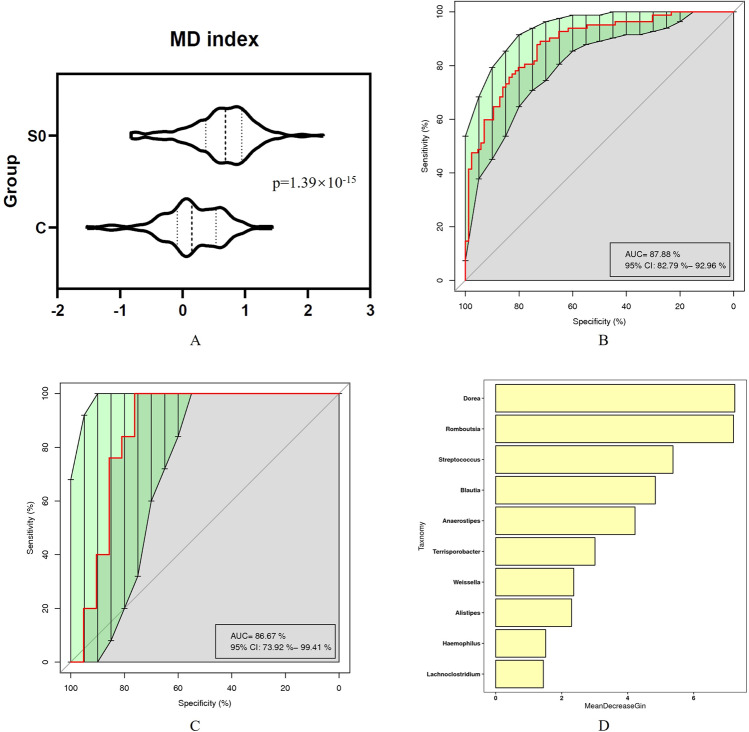
Alteration of microbial biomarkers in SCH patients. **A** Comparison of MD index between SCH patients and HCs. **B** and **C** prediction models for discovery samples **B** and testing samples **C**. **D** The 10 microbial biomarkers in descending order of Gini coefficient. S0: SCH patients, C: healthy controls. FDR were used to correct the multiple testing.

We discovered 19 genera that were significantly different between SCH patients and HCs (all p<0.05). The genus *Lachnoclostridium* showed higher levels and the other 18 genera showed lower levels in SCH patients than in HCs (all *p* < 0.05) (Supplementary Table 3). Of note, 14 of these genera (73.7%) are members of the phylum *Firmicutes*. We observed a significantly higher level of MD index in SCH patients than in HCs (*p* = 1.39 × 10^−15^) (Fig. 3A). We constructed a Random Forest predictive model for SCH patients using the abundance of these 19 microbial biomarkers as predictors. In the discovery set (on average, 82 SCH patients and 86 HCs), a predictive model of 10 microbial biomarkers (*Dorea*, *Romboutsia*, *Streptococcus*, *Blautia*, *Anaerostipes*, *Terrisporobacter*, *Weissella*, *Alistipes*, *Haemophilus*, and *Lachnoclostridium*) showed a remarkable discriminating power, with an AUC of 0.879 (Fig. 3B). In the verification set (on average, 25 SCH patients and 21 HCs), the model effectively differentiated SCH patients from HCs with an AUC of 0.867 (Fig. 3C). These 10 microbial biomarkers are shown in Fig. 3D in descending order of Gini importance.

### Changes of gut microbiota in SCH patients

**Fig. 4:**
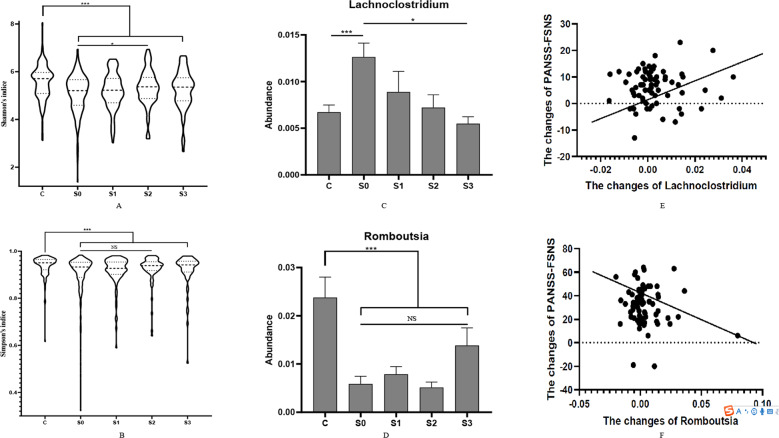
Dynamic changes of gut microbiota in SCH patients. **A** and **B** α diversity, is lower in SCH patients than in HCs (indicated by C) at baseline and was increased after risperidone treatment in SCH patients. **C** and **D** SCH patients show a higher level of *Lachnoclostridium* and a lower level of *Romboutsia* than HCs. **E** and **F** The correlation between the changes of *Lachnoclostridium*, *Romboutsia* and clinical improvement measured by PANSS-FSNS. FDR were used to correct the multiple testing. *p < 0.05, **p < 0.01, ***p < 0. 001.

We observed significant changes in microbial diversity and microbial abundance during the 24 weeks of risperidone treatment. The α-diversity (both Shannon and Simpson’s) indices of SCH patients increased (*p* = 0.046 and 0.057, respectively) after 24 weeks of treatment, but were still lower than the baseline levels of HCs (all *p* < 0.05) (Fig. 4A, B). To identify the effect of risperidone treatment on whole microbiota composition in SCH patients, we used the LEfSe algorithm to compare microbial biomarkers before and after risperidone treatment (Supplementary Figure 5A, B). A total of 14 taxonomies had significantly different levels across different time points. *Lachnoclostridium* was enriched in patients before risperidone treatment. After 24 weeks of treatment, the gut microbiota in patients was dominated by *Lachnospira*, *Sutterella*, and *Alistipes*. Among the 19 microbial biomarkers discovered at baseline, two genera showed changes compared to the baseline levels. Specifically, *Lachnoclostridium* decreased (*p* = 0.019) and *Romboutsia* increased after risperidone treatment (*p* = 0.067) (Fig. 4C, D). At week 24, 14 out of these 19 biomarkers show significantly lower abundance levels in SCH patients compared with HCs (all *p* < 0.05).

### Relationship between microbial biomarkers and clinical symptoms of SCH at baseline

The two genera, *Lachnoclostridium* and *Romboutsia*, and phylum *Firmicutes* [11, 41] were tested for their association with the severity of SCH symptoms at baseline. After controlling for various covariates in partial correlation analysis, the *Lachnoclostridium* abundance was positively correlated with the PANSS-FSNS (*r* = 0.458, *p* = 0.002); the abundance of *Firmicutes* was found to be positively correlated with the PANSS-FSNS (*r* = 0.384, *p* = 0.014). These associations remained to be significant after inflammatory biomarkers and MD index were included as additional covariates.

### Relationship between microbial biomarkers and treatment response

The two genera, *Lachnoclostridium* and *Romboutsia*, were also examined for their associations with the treatment response at week 24. After controlling for various covariates, the changes in *Lachnoclostridium* were positively correlated with the changes in the PANSS-FSNS (*p* = 0.006) (Fig. 4E). The changes in *Romboutsia* (*p* = 0.002) were negatively correlated with the changes in PANSS-FSNS (Fig. 4F). In addition, we found that higher baseline levels of *Romboutsia* and *Lachnoclostridium* were associated with poor treatment response (*p* = 0.006 and 0.005, respectively).

## Discussion

In the present study, we found a significant relationship between microbial biomarkers and clinical response after 24-week risperidone treatment in the first-episode, drug-naïve SCH patients. These findings suggest a role of the gut microbiota in SCH psychopathology and indicate that microbiota measures may serve as biomarkers in SCH treatment.

Our results are consistent with previous findings [5, 9]. Bacterial diversity in the gut measured by Shannon and Simpson’s indices (richness and evenness) is generally thought to be related to health [42], and low α-diversity has been associated with a range of chronic human diseases [43, 44]. Several studies have shown a lower microbial α-diversity in SCH patients than in HCs [5, 9]. Here, we further suggested that low α-diversity is associated with disease severity of SCH, such as positive symptoms. β-diversity presents the similarity or dissimilarity between two or more sets of microbial communities. Consistent with previous reports [5, 12, 20], our study shows that at the community level, SCH patients may have a distinct intestinal ecological state than in HCs [5, 12, 20]. Our findings were also in line with previous reports at several taxonomic levels in SCH patients [45]. However, some other studies found no significant difference in α-diversity between SCH patients and HCs [12, 20, 45]. The discrepant findings may be attributed to the differences in participants (first episode vs. chronic disease phase, antipsychotic medication exposure, geographic locations, and lifestyle).

We discovered 19 genera that showed significant differences between SCH patients and HCs at baseline. Five of them were consistent with the findings from previous studies, i.e., *Streptococcus*, *Haemophilus*, *Enterococcus*, *Blautia*, and *Bifidobacterium* [9, 11, 15, 20, 23]. In addition, the abundance of the *Lachnospiraceae* family was reported to be decreased in SCH [5]. Our study found that six genera (*Blautia*, *unidentified_Lachnospiraceae*, *Anaerostipes*, *Dorea*, *Fusicatenibacter*, and *Butyrivibrio*) that belong to family *Lachnospiraceae* decreased in SCH patients. The remaining nine biomarkers discovered in our study have not been reported previously. Most previous studies have reported the impact of disease duration and antipsychotic medication uses on the composition of gut microbiota [46, 47], our study minimized such confounding effects. We found that microbiota from *Firmicutes* may play an important role in the pathogenesis of SCH and other psychiatric diseases [48–50]. Studies from our group have demonstrated a significant role of immune and inflammatory processes, such as increased levels of hs-CRP and HCY, in the risk of SCH [51, 52]. Both gut microbiota and inflammation have been associated with the pathogenesis of SCH [9]. The dysbiosis of gut microbiota could lead to inflammation in several diseases [53]. We did not find significant correlations between hs-CRP, HCY, and any of the 19 differential genera. This may be attributed to the complex relationships between inflammation and gut microbiota in SCH. Immune dysfunction associated with the changes in the gut ecosystem may not necessarily reflected by the levels of hs-CRP and HCY may by perturbed by changes in gut ecosystems. Future studies are warranted to answer questions, such as whether abnormal gut microbiota causes a dysfunction of the immune response, or whether dysfunction of the immune response affects the gut ecosystems, therefore resulting in an increased risk of SCH.

Our study also proposed a prediction model of 10 microbial biomarkers, which showed high accuracy (AUC = 0.879), and validated in the verification set (AUC = 0.867), indicating a powerful classification model. Previous studies have shown that SCH patients contain harmful microbial species that may contribute to SCH symptoms. Five microbial biomarkers (*Dorea*, *Streptococcus*, *Blautia*, *Anaerostipes*, and *Haemophilus*) in the present study were reported in previous studies [12, 14, 19, 20, 20]. Future studies should further confirm the clinical utility of our predictive model.

Our results suggest an increased α-diversity in SCH patients after risperidone treatment. A recent study in an ex vivo fermentation assay showed that, after 24 h of incubation, risperidone was completely decomposed to ketone, suggesting that microbiota-based biotransformation of risperidone [54]. Notably, we showed that the baseline levels of *Lachnoclostridium*, *Romboutsia*, both belonging to *Firmicutes*, could be used to predict the treatment response. The genus *Lachnoclostridium* was elevated in SCH at baseline and reversed after risperidone treatment. The changes of this genera were positively correlated with the changes in clinical symptoms.

Previous studies have reported the effect of gut microbes on the function of the neurotransmitter systems and amino acid metabolism [5, 10]. The level of *Firmicutes* could impact the function of the dopamine pathway through its modulation on the level of dopamine β-hydrolase [55]. Moreover, low levels of *Romboutsia*, a short-chain fatty acid (SCFA) producing bacteria, were associated with the disturbances of glutamate/GABA-glutamine cycle and astrocyte-neuron metabolism system [55]. In addition, *Lachnoclostridium* has been reported to affect the tryptophan metabolism [56]. Therefore, microbial biomarkers for SCH discovered in our study can affect the function of the neurotransmitter systems and amino acid metabolism as reported in previous studies [57].

The present study has several strengths. SCH patients and HC subjects were demographically matched by age, gender, education, smoking habits, and BMI. Only the first-episode and drug-naïve SCH patients were included in this study. Thus, confounding effects from antipsychotic treatment and disease durations were largely eliminated. We confirmed the stability of our sampling and 16 s RNA gene sequencing protocols by analyzing 28 duplicated samples. In addition, the gut microbiota was tested with the participant status (SCH patients vs HCs) blinded. Furthermore, our study design followed the patients for a relatively longer period than previous studies [45]. However, our study also has limitations. We did not follow the matched HCs. The stochastic fluctuations in the changes of microbial marker levels in SCH patients could not be controlled. However, our relatively larger sample size may overweigh these random noises inherent in the data. We only focused on risperidone treatment of SCH. Therefore, our results may not be generalizable to treatment with other antipsychotic medications.

In conclusion, we characterized potential microbial biomarkers in the first-episode, drug-naïve SCH patients and their role in relation to clinical response to risperidone treatment Our results suggest a role of microbiota in SCH pathobiology. Novel intervention strategies that target microbiota might be promising in schizophrenia treatment.

## Supplementary information

Supplementary Material
