# Supplementary material for: Gut microbial biomarkers for the treatment response in first-episode, drug-naïve schizophrenia: a 24-week follow-up study
Source: Transl Psychiatry. 2021 Aug 10;11:422. doi: 10.1038/s41398-021-01531-3 (PMC8355081; doi:10.1038/s41398-021-01531-3)
Supplement: Supplementary file 1 — Supplementary Material [file 41398_2021_1531_MOESM1_ESM.doc]

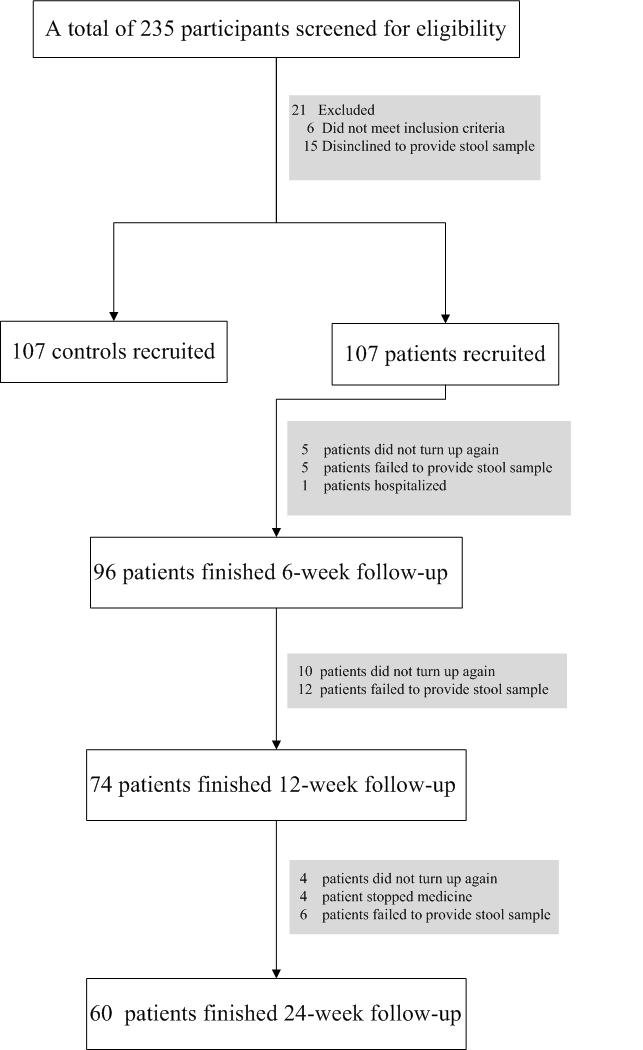


Supplementary Figure 1. The flow chart for the design of the present study. A total of 107 patients with SCH and 107 healthy controls (HCs) were enrolled in the study. Among the 107 patients, all data were re-collected for 96, 74, 60 of them after 6 weeks, 12 weeks and 24 weeks during risperidone treatment. In total, 444 samples were used for further analysis.


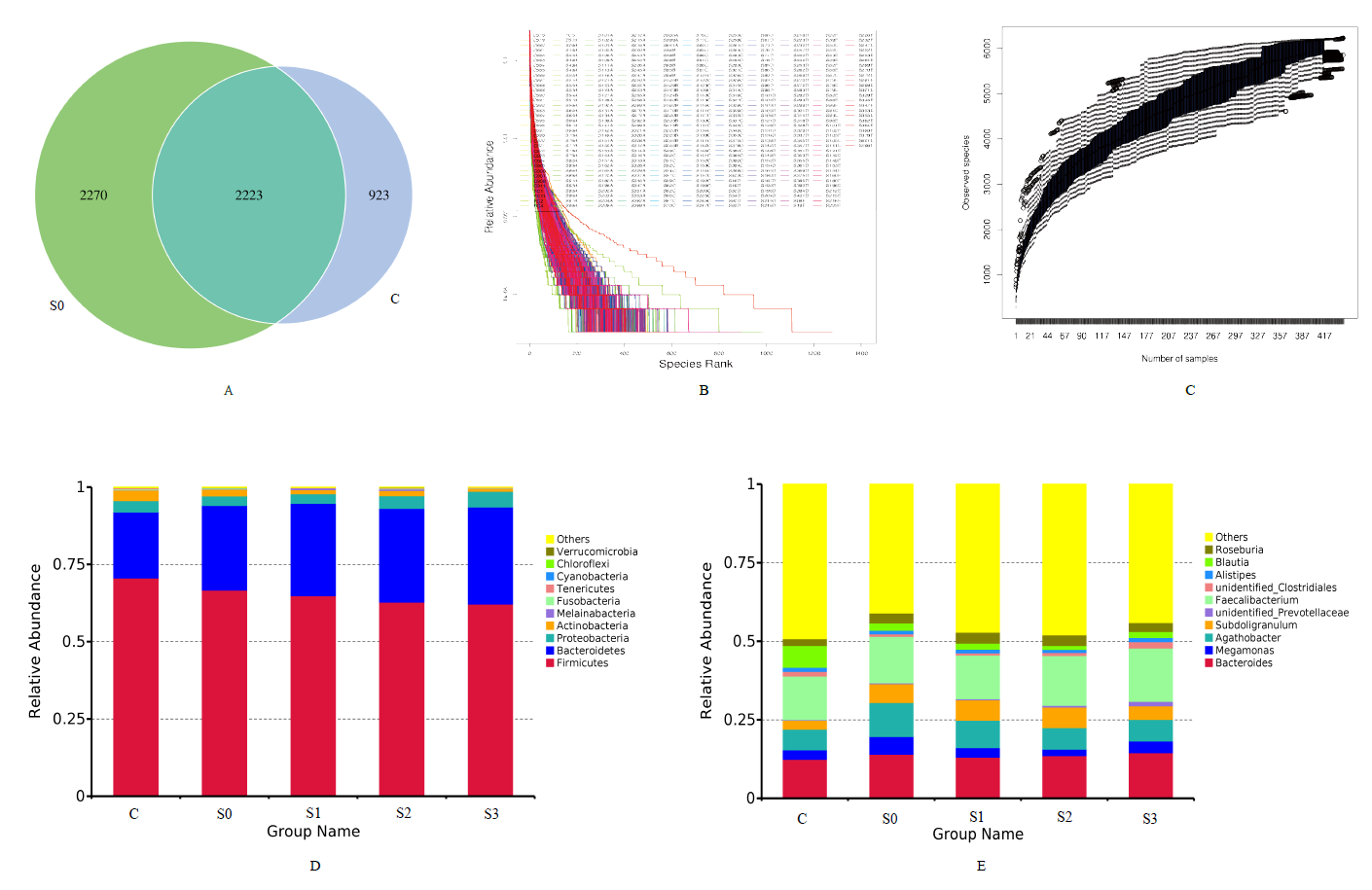


Supplementary Figure 2. OTU analysis between groups. (A) Venn diagram of OTU number in SCH patients and HCs. (B) Rarefaction curves, the abscissa is the number of sequencing lines, the ordinate is the number of OTU obtained. Different samples are represented by curves of different colors. (C) The box plots show the rarefactions approaching the saturation plateau, indicating that sample is sufficient for data analysis; (D-E) The predominant phylum and genus of bacteria among HCs, patients at baseline and follow-ups were show by stacked bar plots. S0: schizophrenia, C: healthy controls.


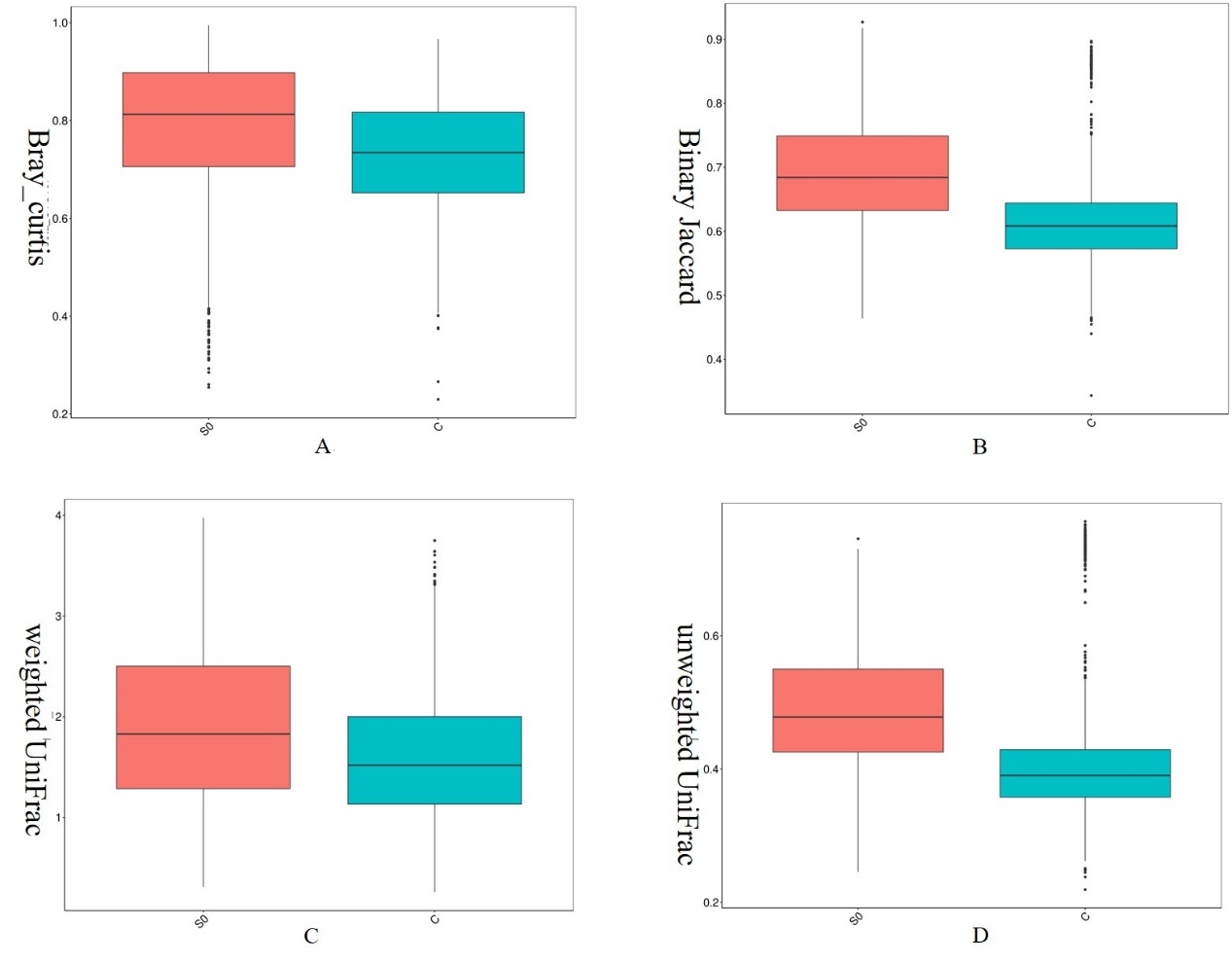


Supplementary Figure 3. The difference of β diversity between SCH patients and HCs. (A-D) The β diversity obtained based on Bray Curtis Dissimilarity (A), Binary Jaccard Dissimilarity (B), Weighted and Unweighted UniFrac Distance (C and D). S0: schizophrenia, C: healthy controls.


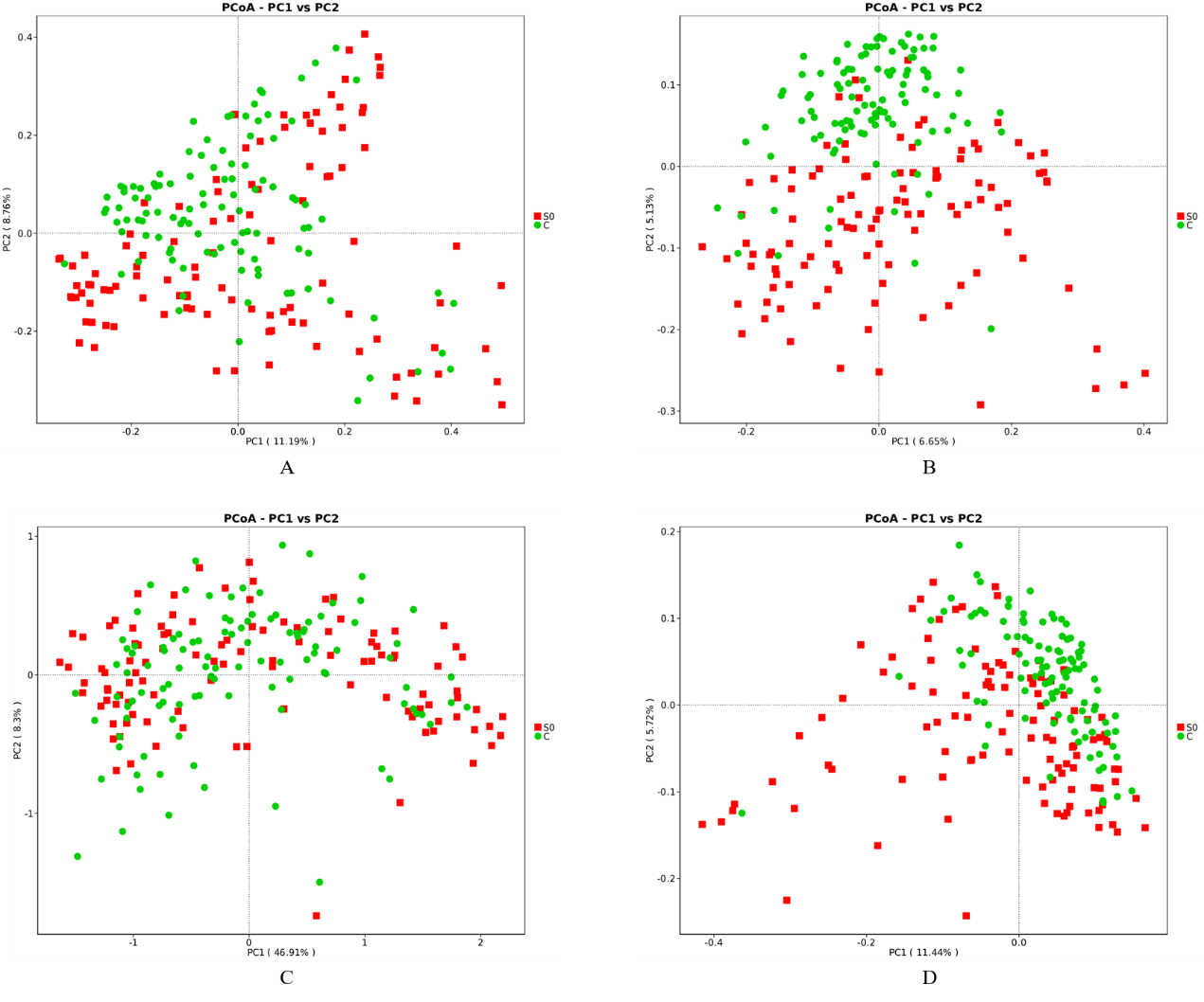


Supplementary Figure 4. The microbial composition difference between SCH patients and HCs. (A-D) The composition of gut microbiota is significant different between SCH patients and HCs in the first two principal components (PC1 and PC2 from the Principal coordinate analyses) based on four β diversity metrics.


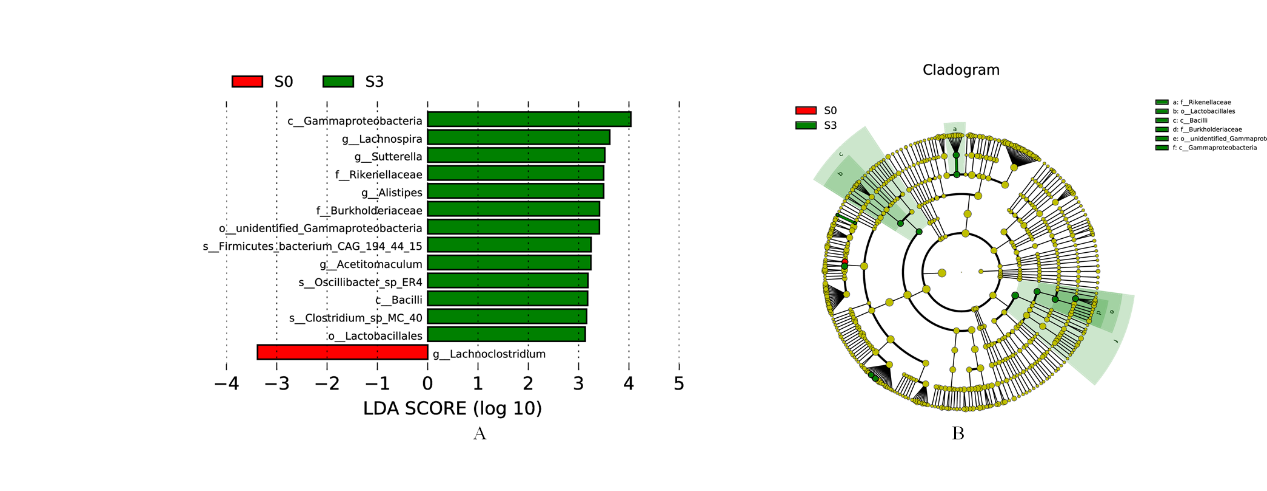


Supplementary Figure 5. The effect of risperidone treatment on microbial composition in SCH patients. (A-B) Taxa enriched in SCH(S0) were shown in red, and those enriched in HCs (C) were shown in green.

| Supplementary Table 1 Paired sample T test of the randomly selected 2 parts of 18 samples | | | | | | | |
| --- | --- | --- | --- | --- | --- | --- | --- |
| Taxonomy | group1, mean (SD), n=28 | group2, mean (SD), n=28 | 95%CI Lower | 95%CI Upper | t value | p |  |
| Bacteroides | 0.135229464286(0.1559362956045) | 0.12852575(0.1509428567688) | -0.08 | 0.09 | 0.163 | 0.872 |  |
| Megamonas | 0.036655125(0.0648102082554) | 0.036534464286(0.0643317683161) | -0.03 | 0.03 | 0.008 | 0.994 |  |
| Agathobacter | 0.07598225(0.0949176553524) | 0.078092678571(0.0902093412639) | -0.05 | 0.05 | -0.089 | 0.93 |  |
| Subdoligranulum | 0.057758178571(0.0905234061408) | 0.064862428571(0.0954798711152) | -0.06 | 0.05 | -0.26 | 0.797 |  |
| unidentified_Prevotellaceae | 0.003755425(0.0109339132886) | 0.006358432143(0.016864490819) | -0.01 | 0.00 | -0.722 | 0.476 |  |
| Faecalibacterium | 0.151767035714(0.0999688407579) | 0.157546714286(0.1035603865585) | -0.06 | 0.04 | -0.234 | 0.816 |  |
| unidentified_Clostridiales | 0.006312821429(0.0091302831) | 0.006837785714(0.0092423006449) | 0.00 | 0.00 | -0.266 | 0.793 |  |
| Alistipes | 0.019516907143(0.0422336005016) | 0.016238964286(0.0395204370479) | -0.02 | 0.02 | 0.354 | 0.726 |  |
| Blautia | 0.02451825(0.025232590445) | 0.023901642857(0.0253734070052) | -0.01 | 0.02 | 0.084 | 0.934 |  |
| Roseburia | 0.035761035714(0.0455808857515) | 0.037333571429(0.0454363241817) | -0.03 | 0.02 | -0.133 | 0.895 |  |
| Bacteroides_fragilis | 0.003640871429(0.0103921077743) | 0.003894364429(0.0102566268881) | -0.01 | 0.01 | -0.086 | 0.932 |  |
| Bacteroides_vulgatus | 0.035237392857(0.0439741936968) | 0.033389101536(0.0430736927152) | -0.02 | 0.03 | 0.155 | 0.878 |  |
| Prevotella_stercorea | 0.002196221429(0.0049407448286) | 0.004155699786(0.0111402769706) | -0.01 | 0.00 | -0.843 | 0.407 |  |
| Bacteroides_plebeius | 0.013872725(0.0227883048676) | 0.014285947536(0.0233708291374) | -0.01 | 0.01 | -0.071 | 0.944 |  |
| Clostridium_disporicum | 0.003259892857(0.0073562551619) | 0.004081032679(0.0081973521221) | 0.00 | 0.00 | -0.522 | 0.606 |  |
| Ruminococcus_bicirculans | 0.008539342857(0.0240299593903) | 0.008605400964(0.0238721980948) | -0.01 | 0.01 | -0.01 | 0.992 |  |
| Bacteroides_stercoris | 0.008457589286(0.0225019110243) | 0.00857856975(0.0223495573368) | -0.01 | 0.01 | -0.019 | 0.985 |  |
| Bacteroides_uniformis | 0.016376339286(0.026792167365) | 0.014291779821(0.0242719582089) | -0.01 | 0.02 | 0.28 | 0.781 |  |
| Clostridium_perfringens | 0.0002116(0.0003848977488) | 0.000214665857(0.0003710181892) | 0.00 | 0.00 | -0.03 | 0.976 |  |
| Ruminococcus_sp_5_1_39BFAA | 0.0128545(0.0163286870546) | 0.011260714286(0.0141054662319) | -0.01 | 0.01 | 0.358 | 0.723 |  |

| Supplementary Table 2. The microbial composition difference between SCH patients and HCs. | | | | | | |
| --- | --- | --- | --- | --- | --- | --- |
| β-diversity dissimilarity metrics | Df | SumsOfSqs | MeanSqs | F.Model | R2 | Pr(>F) |
| Bray-Curtis dissimilarity | 1(213) | 1.897(63.462) | 1.89700(0.29794) | 6.367 | 0.02902(0.97098) | 0.001 |
| Binary Jaccard dissimilarity | 1(213) | 1.424(45.913) | 1.42400(0.21555) | 6.6063 | 0.03008(0.96992) | 0.001 |
| weighted UniFrac distance | 1(213) | 11.45(381.49) | 11.4518(1.7911) | 6.3939 | 0.02914(0.97086) | 0.001 |
| unweighted UniFrac distance | 1(213) | 0.7476(21.9643) | 0.74760(0.10312) | 7.2499 | 0.03292(0.96708) | 0.001 |

Note: Df: degree of freedom; SumsOfSqs: sum of square deviation; MeanSqs: mean square (SumsOfSqs/Df). F.Model: F test value; R2: group square deviation / sum of square deviation.

Supplementary Table 3. The microbial markers difference between SCH patient and HCs at baseline.

|  | Healthy controls | | | Schizophrenia group | | | Wilcoxon W | Z value | p value | adjusted p |
| --- | --- | --- | --- | --- | --- | --- | --- | --- | --- | --- |
|  | mean | sd | SEM | mean | sd | SEM |
| unidentified_Clostridiales | 0.015242 | 0.041078 | 0.003971189 | 0.008389 | 0.032967 | 0.003187 | 9853 | -3.642 | 0.000272 | 0.013577 |
| Alistipes | 0.012594 | 0.020858 | 0.002016463 | 0.011412 | 0.053277 | 0.005151 | 9401.5 | -4.641 | 3.49E-06 | 0.000174 |
| Blautia | 0.069951 | 0.06369 | 0.006157166 | 0.023753 | 0.052334 | 0.005059 | 8187.5 | -7.319 | 2.51E-13 | 1.26E-11 |
| Bifidobacterium | 0.031595 | 0.060263 | 0.005825847 | 0.017074 | 0.032018 | 0.003095 | 9699 | -3.982 | 6.86E-05 | 0.003432 |
| unidentified_Lachnospiraceae | 0.051989 | 0.046558 | 0.004500956 | 0.031995 | 0.037079 | 0.003585 | 9527 | -4.362 | 1.3E-05 | 0.000648 |
| Romboutsia | 0.023767 | 0.043975 | 0.004251187 | 0.005856 | 0.016498 | 0.001595 | 8327 | -7.012 | 2.38E-12 | 1.19E-10 |
| Lachnoclostridium | 0.005709 | 0.008904 | 0.00086081 | 0.010438 | 0.013922 | 0.001346 | 9571 | -4.265 | 2.01E-05 | 0.001006 |
| Fusobacterium | 0.003092 | 0.018131 | 0.001752804 | 0.002514 | 0.012084 | 0.001168 | 9902.5 | -3.68 | 0.000234 | 0.011713 |
| Anaerostipes | 0.014713 | 0.024891 | 0.00240628 | 0.004852 | 0.013362 | 0.001292 | 8520 | -6.586 | 4.56E-11 | 2.28E-09 |
| Streptococcus | 0.012815 | 0.021405 | 0.002069271 | 0.003044 | 0.007818 | 0.000756 | 8345.5 | -6.971 | 3.16E-12 | 1.58E-10 |
| Dorea | 0.014042 | 0.019692 | 0.001903742 | 0.004579 | 0.006667 | 0.000645 | 8224.5 | -7.238 | 4.6E-13 | 2.3E-11 |
| Fusicatenibacter | 0.018005 | 0.024198 | 0.002339289 | 0.005456 | 0.00852 | 0.000824 | 8749 | -6.08 | 1.21E-09 | 6.06E-08 |
| unidentified_Erysipelotrichaceae | 0.012543 | 0.020508 | 0.001982554 | 0.006054 | 0.012795 | 0.001237 | 9473.5 | -4.48 | 7.5E-06 | 0.000375 |
| Lactococcus | 0.002002 | 0.012131 | 0.00117274 | 3.24E-05 | 0.000145 | 1.4E-05 | 8691 | -6.885 | 5.84E-12 | 2.92E-10 |
| Weissella | 0.002979 | 0.011432 | 0.00110517 | 0.000183 | 0.000454 | 4.39E-05 | 8405.5 | -6.996 | 2.65E-12 | 1.33E-10 |
| Terrisporobacter | 0.001562 | 0.003989 | 0.000385595 | 0.000153 | 0.000311 | 3E-05 | 8684 | -6.278 | 3.47E-10 | 1.73E-08 |
| Haemophilus | 0.002612 | 0.008662 | 0.000837368 | 0.001197 | 0.003163 | 0.000306 | 9561 | -4.305 | 1.68E-05 | 0.000839 |
| Enterococcus | 0.002116 | 0.009408 | 0.000909502 | 0.000181 | 0.000792 | 7.66E-05 | 8794.5 | -6.286 | 3.27E-10 | 1.64E-08 |
| Citrobacter | 0.001111 | 0.003143 | 0.000303827 | 0.000415 | 0.001456 | 0.000141 | 9842 | -3.729 | 0.000193 | 0.009671 |
| Note: sd: [standard](javascript:;) [deviation](javascript:;), SEM: standard error of mean, FDR was used to correct the multiple testing. | | | | | | | | | | |
